# 

**Published:** 1908-02-01

**Authors:** 


CONTENTS.

| | PAGE |
|---|---|
| **The Certainties and Uncertainties of Tuberculosis** ... | 461 |
| **The Declining Birth-Rate** ... | 462 |
| **Annotations—** | |
| Medical Women and the Royal Colleges—The Late Sir Thos. McCall Anderson, M.D. — The Ophthalmo-Tuberculin Reaction ... | 463 |
| **Medical Opinion and Movement** ... | 464 |
| **Hospital Clinics—** | |
| Prostatectomy and Bladder Stone. By Reginald Harrison, F.R.C.S. ... | 465 |
| **Special Article—** | |
| Nephritis Associated with Scarlet Fever ... | 466 |
| **Clinical Points—** | |
| Heart-Block ... | 471 |
| **Points in Surgery—** | |
| Psoas Abscess ... | 473 |
| Some Diseases of the Male Genital System... | 474 |
| **Diseases of Children—** | |
| Intestinal Digestion in Babies ... | 475 |
| **Dermatology—** | |
| The Skin in Winter ... | 476 |
| **Gynæcology—** | |
| The Accidents which may Happen to Ovarian Cystic Tumours | 477 |
| **Laryngology and Rhinology—** | |
| Cancer of the Larynx ... | 478 |
| **Otology—** | |
| Concerning Operations for Otorrhœa... | 479 |
| **The Practitioner's Relaxations and Hobbies—** | |
| Miniature Rifle Shooting ... | 480 |
| **Hospital Administration—** | |
| The London Hospital ... | 481 |
| The Hospitals of Paris ... | 483 |
| News and Coming Events ... | 484 |
| **Nursing Administration—** | |
| The Poor-law Midwifery Schools ... | 485 |
| The Graduates' Medical Schools ... | 486 |
| **Hospital Sunday Collections in London in 1907** | 486 |

